# An Intelligent Water Monitoring IoT System for Ecological Environment and Smart Cities

**DOI:** 10.3390/s23208540

**Published:** 2023-10-18

**Authors:** Shih-Lun Chen, He-Sheng Chou, Chun-Hsiang Huang, Chih-Yun Chen, Liang-Yu Li, Ching-Hui Huang, Yu-Yu Chen, Jyh-Haw Tang, Wen-Hui Chang, Je-Sheng Huang

**Affiliations:** 1 Department of Electronic Engineering, Chung Yuan Christian University, Taoyuan City 320314, Taiwan; chrischen@cycu.edu.tw (S.-L.C.); g10776018@cycu.edu.tw (H.-S.C.); g11276031@cycu.edu.tw (C.-H.H.); s10926138@cycu.edu.tw (C.-Y.C.); s10926148@cycu.edu.tw (L.-Y.L.); 2 Department of Interior Design, Chung Yuan Christian University, Taoyuan City 320314, Taiwan; g10882011@cycu.edu.tw; 3 Department of Civil Engineering, Chung Yuan Christian University, Taoyuan City 320314, Taiwan; jyhhaw@cycu.edu.tw; 4 Department of Applied Linguistics and Language Studies, Chung Yuan Christian University, Taoyuan City 320314, Taiwan; 5 Department of Commercial Design, Chung Yuan Christian University, Taoyuan City 320314, Taiwan; jesheng@cycu.edu.tw

**Keywords:** Internet of Things (IoT), machine intelligence, smart city, water level monitoring, water quality monitoring, self-adapting software engineering

## Abstract

Global precipitation is becoming increasingly intense due to the extreme climate. Therefore, creating new technology to manage water resources is crucial. To create a sustainable urban and ecological environment, a water level and water quality control system implementing artificial intelligence is presented in this research. The proposed smart monitoring system consists of four sensors (two different liquid level sensors, a turbidity and pH sensor, and a water oxygen sensor), a control module (an MCU, a motor, a pump, and a drain), and a power and communication system (a solar panel, a battery, and a wireless communication module). The system focuses on low-cost Internet of Things (IoT) devices along with low power consumption and high precision. This proposal collects rainfall from the preceding 10 years in the application region as well as the region’s meteorological bureau’s weekly weather report and uses artificial intelligence to compute the appropriate water level. More importantly, the adoption of dynamic adjustment systems can reserve and modify water resources in the application region more efficiently. Compared to existing technologies, the measurement approach utilized in this study not only achieves cost savings exceeding 60% but also enhances water level measurement accuracy by over 15% through the successful implementation of water level calibration decisions utilizing multiple distinct sensors. Of greater significance, the dynamic adjustment systems proposed in this research offer the potential for conserving water resources by more than 15% in an effective manner. As a result, the adoption of this technology may efficiently reserve and distribute water resources for smart cities as well as reduce substantial losses caused by anomalous water resources, such as floods, droughts, and ecological concerns.

## 1. Introduction

In recent years, the global average temperature has risen by 1.1 °C, and the impacts of climate change are pervasive. A warning report issued by the United Nations indicates that for every 1 °C increase in temperature, the intensity of extreme rainfall events is projected to increase by 7%. However, due to geographical factors, some regions struggle with inadequate water retention, exacerbating the challenges posed by alternating floods and droughts. Meanwhile, population growth and industrial expansion have heightened the demand for water resources, underscoring the critical need for effective management of limited resources [1].

Statistics show that only 0.03% of freshwater resources on Earth are available for human use [2,3,4]. Storing freshwater is crucial, and efforts have been made to establish reservoirs, dams, and other storage facilities in areas naturally conducive to water storage [5]. Yet, due to the uneven distribution of water resources [6], some countries are prone to floods while others face severe droughts which also lead to food crises [7,8]. Globally, at least 80 countries experience arid or semi-arid conditions, and around 40% of the global population is affected by concurrent drought [9,10,11].

Climate change not only exacerbates the frequency of extreme weather events but also magnifies the challenges of water resource management. Intense rainfall during the rainy season leads to widespread flooding, while drought periods cause reservoir levels to drop as well as water scarcity, adversely affecting agriculture and livelihoods [12,13]. Therefore, water resources are crucial for human survival and ecological health, sustaining fertile soil, promoting plant growth, and playing a critical role in environmental purification, fire prevention, and energy production. Masud, M.B et al. explored the relationship between water footprint and crop yields [14], and the issue of water management becoming a major global challenge is addressed in other reports as well [15]. Thus, effective water resource management is vital for economic development and social well-being. However, contaminated water sources pose significant health and environmental risks, emphasizing the importance of protecting and conserving water resources. This is the reason why, in recent papers, many researchers use machine learning to train past statistics into models suitable for future predictions [16,17,18].

Against the backdrop of escalating global climate change, the development of innovative water resource management technologies becomes crucial. Therefore, this paper proposes an automated real-time monitoring system for multiple ponds using Internet of Things (IoT) technology [19,20]. This system integrates IoT techniques, achieving low-cost, low-energy, and high-precision operation through various sensors, control modules, and power communication systems [21,22]. Farmanullah Jan et al. proposed a system including turbidity, pH, temperature, and conductivity sensors and data transferal via an ESP8266 Wi-Fi module [23], which could build a small but robust water quality monitoring system to sense water quality data and transmit the data wirelessly. The research refers to the architecture mentioned above and uses artificial intelligence to analyze historical rainfall data and weather forecasts. The system can dynamically adjust water levels, reducing costs, enhancing measurement accuracy, and offering a promising solution for effective water resource preservation.

## 2. Methodology

**Figure 1:**
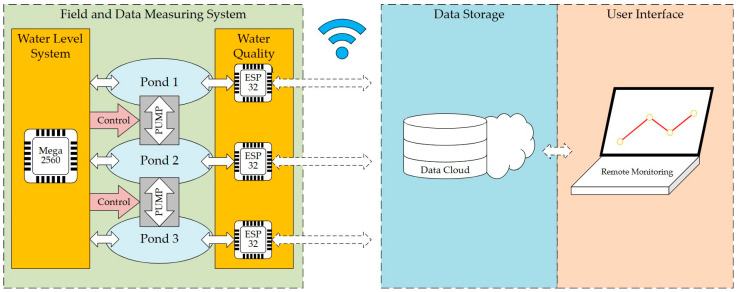
Proposed real-time monitoring system.

The proposed water monitoring IoT system is a cutting-edge monitoring solution, equipped with a turbidity, oxygen, and water level sensor system. This monitoring system is specifically designed for use with ecological wetlands. Through precise calculations using multi-step measuring methods, the proposed system minimizes the impact of extreme readings and enhances overall accuracy. The data obtained by the sensors are connected to the cloud database through ESP-32’s Wi-Fi function and are presented as clear line graphs as Figure 1. Additionally, the system features warning notifications, allowing users to intuitively understand water quality and assisting users in the data-driven analysis of eco-friendly purification effectiveness, promoting environmental education.

### 2.1. Field and Data Measuring System

The field and data measuring system was set up in different ponds. The system comprised three core components, delineated as follows:

(1) Aquatic environment monitoring sensors: These sensors encompass a range of devices designed to measure water quality parameters such as pH, turbidity, temperature, and dissolved oxygen, as well as water level indicators. They were strategically positioned within each pond to facilitate real-time monitoring of the prevailing aquatic conditions;
(2) ESP-32 node for data reception and transmission: The ESP-32 module played a pivotal role in the system’s functionality. Its primary responsibility was to serve as a node that receives sensor data. Data transmission occurred through a physical wired connection to ensure data integrity and reliability. The ESP-32 acted as a data intermediary, forwarding the collected information to the central control unit for processing and management;
(3) Mega 2560 control unit and pump mechanism: At the heart of the system lied the Mega 2560 control unit, which controlled the pump mechanisms. Through a sophisticated control algorithm, the Mega 2560 assessed the incoming sensor data and made real-time decisions regarding pump activation and water management strategies. This centralized control unit ensured optimal water quality and level maintenance in each pond.

In the context of each pond, these sensors collaborated to provide continuous monitoring of water quality and level parameters. The collected data were transmitted through physical connections to the ESP-32, which acted as a pivotal data hub. Furthermore, the ESP-32 employed Wi-Fi connectivity to relay this information to cloud-based servers, ensuring that the system remained updated with the latest data trends and enabling remote monitoring and management capabilities.

#### 2.1.1. Aquatic Environment Monitoring Sensors

This study used various sensors for water quality detection, with basic testing items including pH, turbidity, hardness, etc. The proposed system selected more critical items for irrigation or drinking water, namely turbidity and DO oxygen content detection [24,25]. These pH sensors were designed to measure the acidity or alkalinity of a solution by quantifying the concentration of hydrogen ions (H+) present in it. By measuring the voltage, the sensor was able to transfer the voltage value into a pH value by the following formula:

(1) $$pH=\frac{AnalogValue\times 5\times 3.5}{1024\times 6}$$

**Figure 2:**
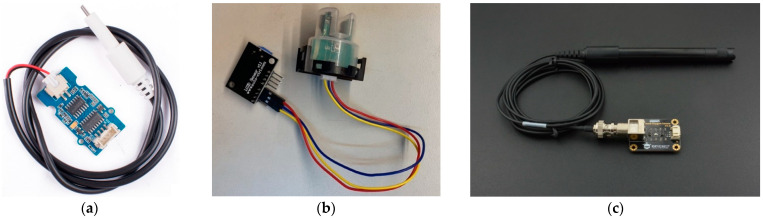
(**a**) pH sensor (**b**) water turbidity sensor (**c**) water dissolved oxygen sensor.

The importance of pH measurement lies in its widespread use across fields such as biology, agriculture, and wastewater treatment, where precise control and monitoring of pH levels are critical for optimal performance and outcomes. The sensor proposed in this paper is shown as Figure 2a.

Turbidity refers to the degree of light scattering in water and is related to the presence of fine organic matter, microorganisms, or plankton in the water [26,27,28]. High turbidity impedes light penetration, affecting the photosynthesis of aquatic plants. By measuring the voltage, the sensor was able to transfer the voltage value into a pH value by the following formula:

(2) $$V=\frac{AnalogValue}{1024}\times 5000\;(mV)$$

(3) $${Turbidity(NTU)=-1120.4V}^{2}+5742.3V-4352$$

Incorporated within the sensor was a dual-tube setup. The transmission of light through a specific volume of water is contingent upon the water’s degree of contamination. Greater water impurities result in diminished light transmission. Turbidimeters can measure water’s transparency and scattering rate to obtain turbidity values, measured in Nephelometric Turbidity Units as shown Figure 2b.

Oxygen in water mainly comes from atmospheric dissolution and photosynthesis by aquatic plants [29,30]. By measuring the voltage, the sensor was able to transfer the voltage value into a pH value by the following formula:

(4) $$DO\left(\frac{mg}{L}\right)=5717.4V-583.33$$

If water is contaminated with organic matter, microorganisms consume dissolved oxygen while breaking down these organic substances, possibly causing respiratory difficulties for aquatic life and even threatening survival as shown in Figure 2c.

In the water quality module, after the raw data were successfully collected by different sensors through the ESP-32, the data were uploaded to a data cloud and stored in case of need. Furthermore, if the water quality is below the standard value, the user receives a system notice through the application. If not, the cycle keeps running. The water quality details refer to Taiwanese class III surface water quality standard values in Table 1.

**Figure 3:**
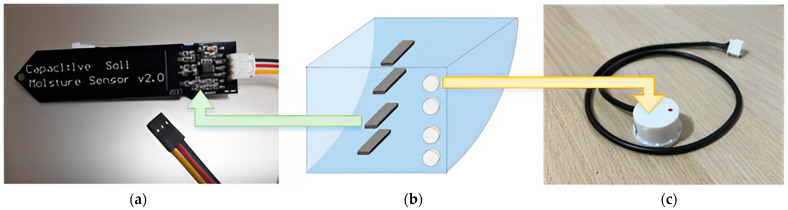
(**a**) Part of the water level monitoring sensor kit. (**b**) The condition in which the water level sensors are settled. (**c**) Liquid level sensor.

There are various sensors available in the market for detecting water level heights, including those implementing ultrasonic [31], magnetic, conductivity, and capacitive methods. The choice of sensor type is contingent upon the specific environment and type of liquid. In this study, the system was required to be installed outdoors without disrupting the original natural environment, necessitating waterproof and dustproof features. Our design objective was to exploit a low-power, low-cost monitoring system, taking into account the installation environment. Therefore, we opted for a low-cost capacitive soil moisture sensor to be used as a liquid surface sensor as shown in Figure 3a. This capacitive soil sensor operates at a voltage between 3.3V and 5V, making it suitable for microcontrollers, fulfilling our design criteria. The principle behind capacitive sensors involves two metal plates and measuring the change in capacitance between the plates when they come into contact with a liquid surface. After observing the highest level and the lowest water level in the wet and dry seasons, we separated the water level into four parts to detect accurate water levels as shown in Figure 3b.

The non-contact liquid level sensor shown in Figure 3c is an advanced detection device, specifically designed to monitor the presence of liquid in a container without directly contacting the liquid. As such, this sensor offers significant advantages, especially when dealing with liquids that have corrosive properties or other specific chemical characteristics. Because of its non-contact nature, the sensor does not need to be submerged in liquids, which prevents damage or wear resulting from prolonged exposure to corrosive liquids, such as strong acids, alkalis, or other erosive materials. Compared to traditional sensors that require direct contact with liquids, the non-contact liquid level sensor is not influenced by the pH, impurities, or floating particles in the water. This ensures the accuracy and reliability of its detection results, making it suitable for various application environments. Additionally, this type of sensor is usually designed with an adjustable knob, allowing users to tailor its detection sensitivity based on specific application needs. It can also detect liquid levels through non-metallic materials, including acrylic, ceramic, and glass. The non-contact liquid level sensor offers an efficient, reliable, and highly adaptable method for monitoring liquids in various containers.

In the water level module, this paper proposed an interval water level that represented four conditions as shown in Table 2. It delineated the region-level conditions as indicated by the sensor values. In instances where equilibrium was required among the water bodies, priority was given to draining water from bodies with higher water levels to those with lower levels. The water level data were uploaded to the cloud through the Mega 2560 board for subsequent data analysis. And the MCU judged whether the water level was too high or too low. If the water was too high, the bump was ordered to turn on to transport the water to other ponds to soothe the flood and vice versa.

**Figure 4:**
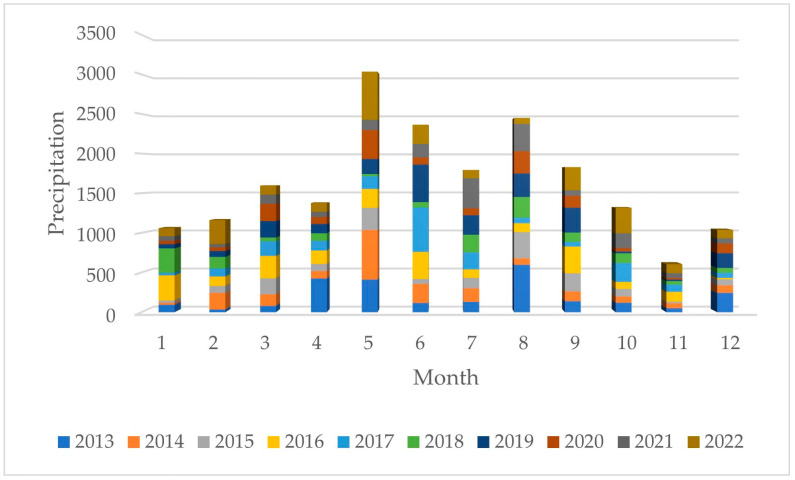
The Taoyuan monthly rainfall from 2013 to 2022.

In this study, we utilized rainfall data from various monthly observations at a rainfall monitoring station (Figure 4). The local rainfall season can be divided into a wet season and a dry season based on monthly rainfall over the past ten years. For the modeling part, the sensor heights were set at 20%, 30%, 70%, and 80% of the pool depth to adapt to different rainfall thresholds during different periods. The peak values during the wet season were set between the region’s levels 3 to 5, while peak values during the dry season were set between the region’s levels 2 to 4.

The capacitive soil moisture sensor’s value can be affected by environmental factors such as temperature and humidity [32]. To prevent these environmental influences from rendering pre-set thresholds ineffective, it is necessary to periodically calibrate the sensor. However, regularly adjusting the manually using the following formula can be cumbersome for users in managing the system:

(5) $$Threshold\;Valu=\frac{\sum_{i=n-15}^{n}{Sensor\;Response\;(Dry)}_{i}+\sum_{i=n-15}^{n}{Sensor\;Response\;(Wet)}_{i}}{30}$$

**Figure 5:**
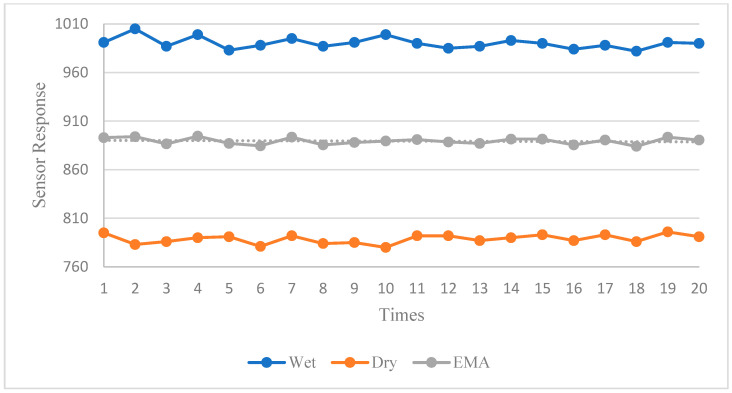
The sensor response and its linear regression.

This study employed machine learning algorithms to continuously update the sensor’s thresholds in real time. The machine learning algorithm utilized statistical analysis of the sensor readings from both above and below the liquid level for the first 15 readings from each sensor. After dynamically regressing the data, it calculated the optimal threshold value for that specific sensor and the original value, and the linear regressions are shown in Figure 5.

**Figure 6:**
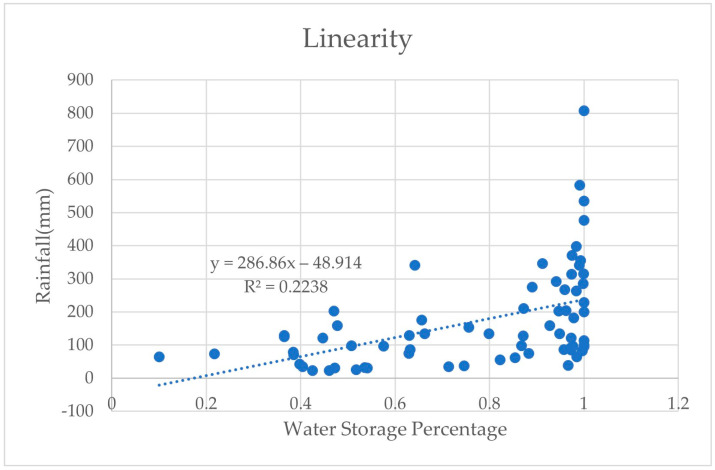
The linear regression graph of rainfall and reservoir water storage.

Based on the changes in rainfall and reservoir water levels in the past 68 months, this study was able to generate a relationship between future rainfall and water levels through linear regression. Linear regression is commonly used to predict and estimate related values. C. Gnaneswar Raju et al. compare linear regression and logistic regression for ground water level detection [33]. Figure 6 shows the linear regression graph of rainfall and reservoir water storage. The dotted line is the relationship line of the correlation coefficient, and each point is a relationship point between the water storage percentage of the month and the rainfall of the month. When the monthly rainfall exceeded 500 mm, the percentage reached 100%. In addition, the increase or decrease in the percentage of water storage had indirect effects through surface runoff, infiltration, etc. Therefore, changes in water levels and rainfall could only be roughly estimated.

#### 2.1.2. ESP-32 Node for Data Reception and Transmission

Since the ponds were widely spread around Taoyuan city, setting many sensors and wires linked to the observatory was considered a useless and expensive way to monitor the ponds. Instead, using a remote method to receive the information from each pond was deemed an excellent approach. The ESP-32 is a 32-bit system-on-chip that combines Wi-Fi and Bluetooth with external flash memory. It includes dual cores and supports Arduino open architecture and can be widely used in various IoT applications. Its most powerful functions are its network functions and module functions. Additionally, the ESP-32, as a low-cost MCU, could be widely spread as a node for each pond. With wireless connection, it was able to upload the water quality data from each pond to the cloud precisely.

#### 2.1.3. Mega 2560 Control Unit and Pump Mechanism

In addition to segregating water quality monitoring data from water level monitoring and control systems, this comprehensive system implemented a highly sophisticated approach to optimizing pond management and ensuring the well-being of aquatic environments. The Mega 2560, acting as the central hub for data aggregation, orchestrated the seamless collection of water-level data from individual pond-level sensor systems. This centralized processing hub formed the nerve center of the entire network, providing real-time insights into water level conditions across various ponds. Expanding on its capabilities, the system deployed a bidirectional water flow control system between interconnected ponds.

The innovative design consisted of two pumps, enabling a dynamic response to changing water level dynamics. When the Mega 2560 detected the need for water level adjustments, it initiated a rapid and precise signaling process. These signals activated the water flow control system, a critical component of the system’s intelligent infrastructure. This dynamic system recalibrated water levels to establish and maintain equilibrium, ensuring that each pond operated optimally within predetermined parameters. Meanwhile, water level information was uploaded to the cloud via the ESP8266 Wi-Fi development board integrated within the Mega 2560.

Due to the necessity of monitoring water level sensors in different ponds and subsequently controlling the operation of pumps between each pond, multiple I/O pins were required for receiving and transmitting water level information and pump control signals, as illustrated in Table 1. It was determined that the ESP-32 alone could not adequately handle the volume of data associated with the entire system. Therefore, the water quality nodes employed the ESP-32 for data collection and transmission, while the water level system consolidated all signals and control pins within the Mega 2560. The Arduino Mega 2560 is a microcontroller development board with 54 digital pins and 16 analog pins for use; its working voltage is 7~12 volts, and the USB interface provides power or uses a power stabilizer and an external battery power supply. The use of the C language development environment is convenient and easy to elaborate upon, and the highly variable characteristics can be widely used in various fields, as shown in Table 3. After comparison with other boards, the Mega 2560 board was selected for main development while the ESP-32 was selected for and could be widely spread in many ponds with low-cost expenses [34].

### 2.2. Data Storage

Data collection was a crucial stage in this system. To ensure more accuracy during data gathering, we utilized multi-measurement methods, i.e., using multiple identical sensors dispersed in the same target area, averaging the results, and ultimately transmitting the data to the ESP-32 microcontroller to upload it to the cloud through Wi-Fi. The upload time interval was adjustable, and the proposed system was set to upload the data every 5 min. Water quality and water level data collection, coupled with ecological surveys, are fundamental components of environmental monitoring and management. Accurate and comprehensive data are critical for assessing the health of aquatic ecosystems and ensuring the safety of water resources for both human consumption and ecosystem sustainability. Concurrently, monitoring water levels provides essential insights into hydrological patterns, seasonal variations, and potential flood risk assessment. Related storage data can be widely used and are essential in making evidence-based decisions and maintaining the health of aquatic ecosystems in the long run.

### 2.3. User Interface

Through the utilization of successfully collected data, Thingspeak offers an intuitive and interactive interface, empowering users to visualize data in diverse formats, such as charts, graphs, and heatmaps [35,36,37]. This visual representation simplifies data interpretation and facilitates the identification of trends, anomalies, and critical insights. In addition, Thingspeak provides a robust Application Programming Interface (API) that simplifies data retrieval and integration with external applications, making it an ideal choice for users seeking to harness the data for further analysis or integration into custom applications. In essence, this data transmission and visualization process not only enhanced the accessibility of our collected data but also significantly improved the overall user-friendliness and applicability of the system. It transformed data into actionable insights, ultimately contributing to decisions and various applications, ranging from environmental monitoring to industrial automation.

**Figure 7:**
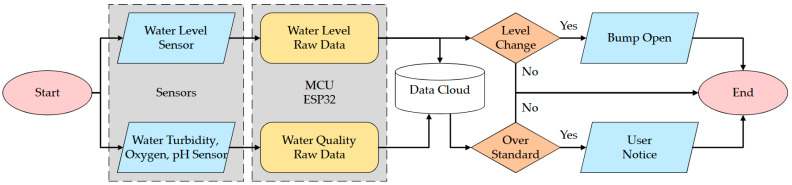
Proposed system flowchart.

The detailed workflow is shown in Figure 7. Our system was mainly divided into two parts: the water quality monitoring module and the water level monitoring module. In the water quality module, after the raw data were successfully collected by different sensors through the ESP-32, the data were uploaded to the data cloud and stored in case of need. Furthermore, if the water quality was below the standard value the system set up, the user received a system notice through an application. If not, the cycle kept running. In the water level module, when the water hit the numbered sensor, the system performs two actions according to the following: First, the data were analyzed through the Mega 2560 board and it was judged whether the water level was too high or not. If the water was too high, the bump which transports the water to other ponds turned on automatically to soothe the flood. Second, the relative water level was uploaded synchronously to the cloud for subsequent data analysis.

## 3. Application Field and Result

**Figure 8:**
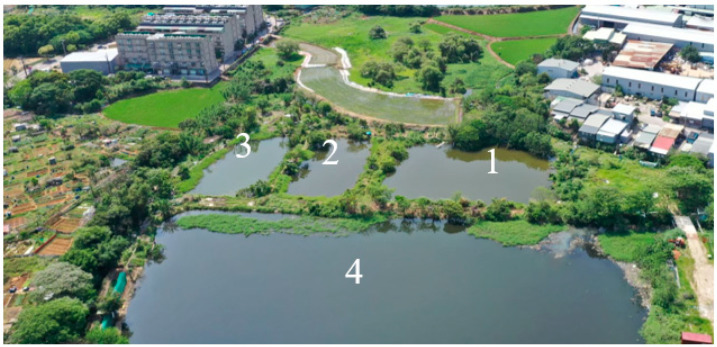
The actual aerial shot of the ponds. The actual aerial shot of the ponds and 1 to 4 in the figure represent waters No. 1 to No. 4 respectively.

In this study, the water level control system was developed based on actual measurements taken from a pond located in Taoyuan City, Taiwan, as shown in Figure 8. The application field consists of a total of four distinct bodies of water, as indicated in the diagram. Each of these bodies of water possesses the capability for mutual regulation, and their water quality must be maintained within recommended parameters.

### 3.1. Water Level Model Making

**Figure 9:**
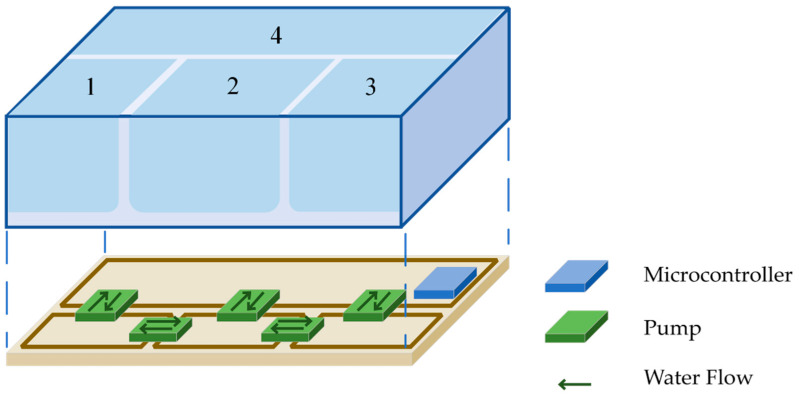
Model design of the real scenario. Model design of the real scenario and 1 to 4 in the figure represent waters No. 1 to No. 4 respectively.

Due to the potential interference that the system may encounter in its practical field implementation, this article establishes a scaled-down proportional model based on the actual field conditions to validate the system’s operation and functionality. After the completion of the prototype, the system will be deployed in the field for real-world application. A scaled-down acrylic model (70 cm × 70 cm × 34 cm) was fabricated to simulate real-world water level control scenarios in natural environments. The model comprised four adjacent water pools of varying sizes. Soil moisture sensors were installed at four equidistant heights in each pool, defining four water level standards from bottom to top. The microcontroller and motor were positioned directly below the pool model, as depicted in Figure 9.

#### 3.1.1. Water Level Simulation Model and Sensors’ Waterproof Measures

**Figure 10:**
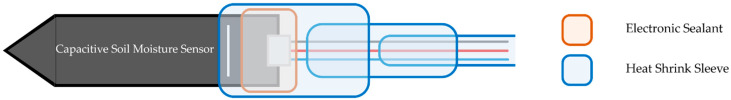
Waterproof demonstration of the water level sensor.

The main structure of the model consists of an acrylic tank with a 5 mm thickness. The design uses polycarbonate sheets in a multi-layered assembly to create differentiated depths in individual water pools. This approach offers a complex and versatile environment that allows for greater variability in testing conditions. To prevent water from leaking through the pipes to the lower level containing the electrical circuits, causing short circuits, multiple layers of elastic cement paint are applied as shown in Figure 10. This specific type of paint is chosen over standard waterproof paints due to its superior adhesive properties and enhanced water resistance. It serves as a robust barrier, effectively preventing water or moisture intrusion. Furthermore, for additional waterproofing assurance, acid silicone fills crucial points, namely at the base of the sensors, at the connection points between the higher and lower layers, and inside the connecting pipes. This acid silicone adds an extra layer of security, making the entire assembly doubly safeguarded against water leaks. By adopting such a comprehensive approach to design and waterproofing, the model ensures not only functional effectiveness but also the critical safety of the lower layer’s electronic circuits.

The intricacy of the pool model extends to its sensory components as well. Each pool in the model is equipped with four soil moisture sensors designed to be submerged underwater for long durations. This submersion makes the waterproofing of these sensors and their connecting wires an imperative aspect of the design. For the electronic components at one end of these sensors, electronic sealant is used as a primary protective layer. This serves as the first line of defense against water ingress and is particularly useful for safeguarding the sensitive electronic parts from moisture and potential short circuits. However, the task of waterproofing and wire management presents a challenge due to the disparity in width between the sensors and the connecting wires. To resolve this, this study employs a tiered approach, making use of both insulating tape and heat-shrink tubing. The tubing and tape are applied in multiple layers and varying sizes, from largest to smallest. This allows the heat-shrink tubing to conform more closely to the shape and size of both the sensors and the connecting wires, thereby ensuring a more secure and watertight fit. This study provides a comprehensive waterproofing solution, adding yet another layer of reliability and effectiveness to the overall model design.

#### 3.1.2. System Configuration

**Figure 11:**
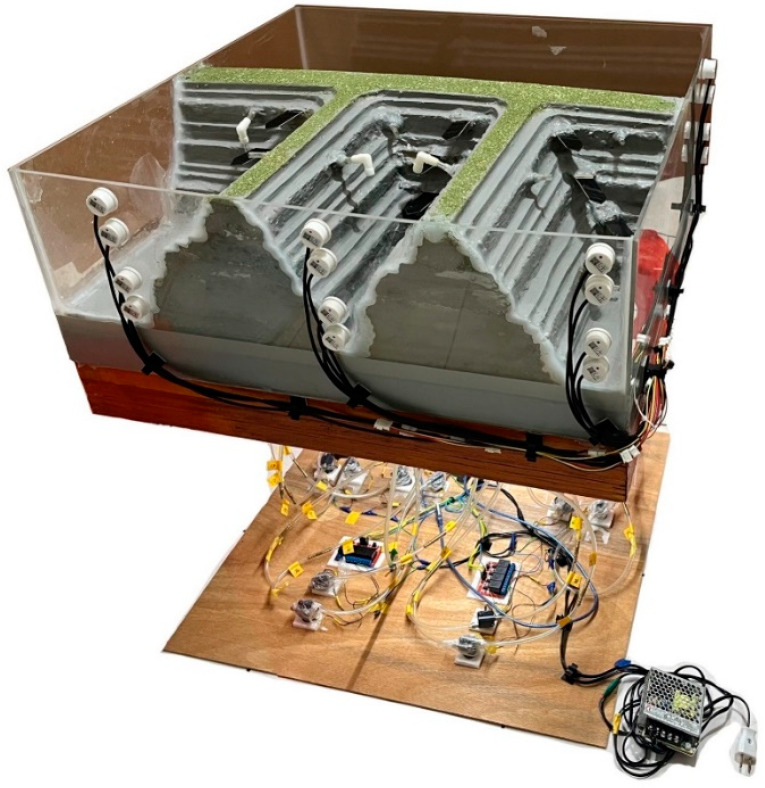
The model of water level monitoring and control system.

The arrangement of circuitry on the baseboard is designed to align with the positions of the pools above, simplifying the placement of relays and motors as Figure 11. Due to the large number of components, clear organization of the wiring is crucial. To address this, this study implements several measures. Wires from the upper layer are bundled together using heat-shrink tubing. This creates a single, manageable wire bundle, making it simpler to trace individual wires and reducing the risk of tangling or misplacement. For the physical interconnection between the upper and lower layers, multi-pin connectors with locking mechanisms are utilized. This design choice facilitates easy detachment and reattachment of the two layers, thus offering a streamlined approach to both regular maintenance and any future adjustments. This study not only simplifies the task of wiring management but also introduces an element of modularity. This makes ongoing maintenance and potential upgrades more efficient, without sacrificing the integrity or functionality of the overall system.

### 3.2. Water Quality Model Making

The model is designed for remote real-time monitoring. The ESP-32 module is used for Wi-Fi connectivity to receive cloud water quality data, keeping users updated about the status on site. Furthermore, the system is programmed to trigger alerts based on pre-set parameters, reminding users to take timely action if any water quality issues are detected, making it convenient for remote water quality monitoring with straightforward data presentation.

**Figure 12:**
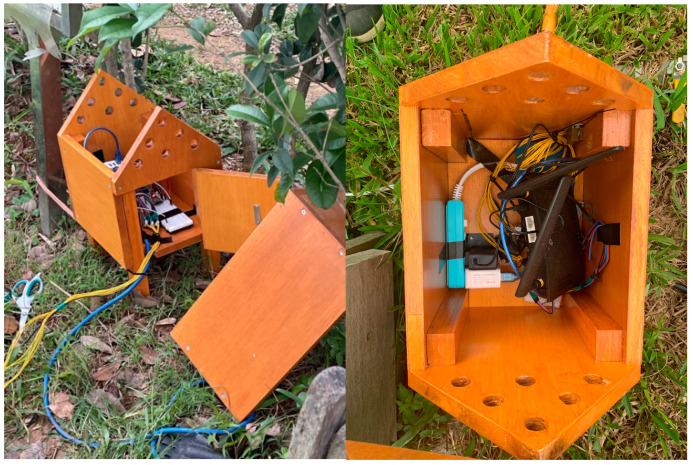
Actual water quality observation model.

The sensors located in the ponds are housed in a small monitoring house made of wood as shown in Figure 12. The cabin is painted with multiple layers of outdoor wood protection paint to withstand environmental conditions. The small house incorporates magnetically attached doors, allowing easy access to the internal components for maintenance purposes. For heat management, the cabin features a spacious, movable eave and ventilation holes strategically placed in the roof section. This design facilitates effective heat dissipation and ensures that the electronic devices within remain at optimal temperatures. The electronic components of the sensors submerged in the water are encapsulated in electronic sealant. This provides an additional layer of waterproofing, making the devices well-suited for prolonged underwater operation without the risk of water infiltration.

Each pond is equipped with one pH sensor, one water turbidity sensor, one water dissolved oxygen sensor, an ESP-32, four soil moisture sensors, and four liquid level sensors. For extension, ponds between ponds can be equipped with pumps, and a Mega 2560 board is installed regardless of the number of nodes. System expansion and details are shown in Table 4; the installation time of a node is approximately one hour.

Furthermore, compared with the another system [38] shown in Table 5, ours not only has more functions, such as water turbidity and water level sensors and an adjustment system, but also reduces the cost by 52.27%; moreover, the functions can be customized to correspond to the requirements, which can be added if necessary.

### 3.3. Configuration and Algorithm

**Figure 13:**
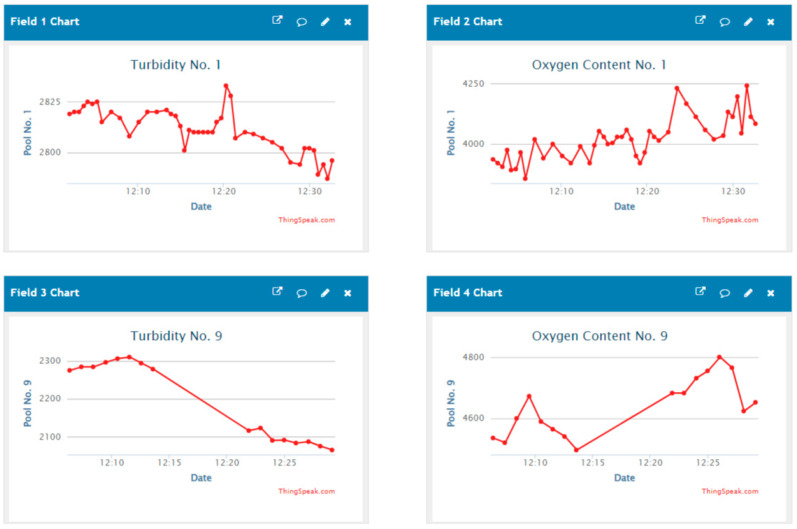
Data line graphs which are uploaded to the cloud.

After the system which can monitor the conditions of sewage and clean water pools in real time has been successfully installed in actual fields, we can obtain data from these MCUs and draw line graphs from the data, which demonstrate the water turbidity and water oxygen of different two ponds as shown in Figure 13. The horizontal axis shows the time, while the vertical axis shows the data on water quality and water level. Each point is spaced at thirty-second intervals adjustable by the user.

## 4. Conclusions

This study is dedicated to the fusion of environmental protection and ecological education, creating a highly integrated intelligent water resource management solution. Our study initially uses a scaled-down model to demonstrate a smart city’s water level monitoring system and automatically adjusts water levels using big data technology. It aims to optimize water resource use and conservation under the impact of climate change. Secondly, our study collaborates with local ecological farms for hands-on implementation, installing real-time water quality monitoring systems that provide not only visualized real-time data but also promote best practices in smart water management. Lastly, through ESP-32 technology, all collected data are uploaded to cloud storage for remote viewing and further big data analysis. This allows the system to be scalable and sets a foundation for future integration of other monitoring points. This comprehensive plan offers a forward-thinking integrated water resource management solution on both local and global levels; moreover, the impact on environmental protection and ecological education could also be shown worldwide.

## Figures and Tables

**Table 1:** Class III surface water quality standard values.

pH Value	Turbidity	Oxygen
6.0~9.0	3.1~40 NTU	Above 4.5 mg/L

**Table 2:** The dry and wet conditions of each numbered sensor.

Region’s Level	Status Description
5	Fully
4	Sufficient
3	Normal
2	Less
1	Lack

**Table 3:** MCU Comparison.

Type	Arduino UNO	Arduino Mega 2560	ESP-32
Microcontroller Chip	ATmega328	ATmega2560	Tensilica 32-bit
Operating Voltage	5 V	5 V	3.3 V
Input Voltage	7–12 V	7–12 V	7–12 V
Digital I/O	14	54	28
Analog Input	6	16	8

**Table 4:** List of costs required for proposed system in one pond.

Items	Amount	Each Price	Total Price
pH Sensor	1	$40	$40
Turbidity Sensor	1	$15	$15
Dissolved Oxygen Sensor	1	$20	$20
Soil Moisture Sensor	4	$4	$16
Liquid Level Sensor	4	$6	$24
ESP-32	1	$6	$6
Mega 2560	1	$12	$12
Pump	2	$7	$14
			$147

**Table 5:** The comparison of function and cost with another system.

Function	[38]	Ours
pH Monitoring	◯	◯
Dissolved Oxygen Monitoring	◯	◯
Temperature Monitoring	◯	◯
Turbidity Monitoring	X	◯
Water Level Monitoring	X	◯
Water Level Control	X	◯
Price	$308	$147

◯ means the function can work X means the function can not work.

## Data Availability

Data Availability Statement: Some or all data, models, or code that support the findings of this study are available upon reasonable request from the corresponding author.
